# Zinc in Cardiovascular Functions and Diseases: Epidemiology and Molecular Mechanisms for Therapeutic Development

**DOI:** 10.3390/ijms24087152

**Published:** 2023-04-12

**Authors:** Takafumi Hara, Emi Yoshigai, Takuto Ohashi, Toshiyuki Fukada

**Affiliations:** Molecular and Cellular Physiology, Faculty of Pharmaceutical Sciences, Tokushima Bunri University, Tokushima 770-8514, Japan

**Keywords:** zinc transporter, zinc signal, cardiovascular disease

## Abstract

Zinc is an essential trace element that plays an important physiological role in numerous cellular processes. Zinc deficiency can result in diverse symptoms, such as impairment of the immune response, skin disorders, and impairments in cardiovascular functions. Recent reports have demonstrated that zinc acts as a signaling molecule, and its signaling pathways, referred to as zinc signals, are related to the molecular mechanisms of cardiovascular functions. Therefore, comprehensive understanding of the significance of zinc-mediated signaling pathways is vital as a function of zinc as a nutritional component and of its molecular mechanisms and targets. Several basic and clinical studies have reported the relationship between zinc level and the onset and pathology of cardiovascular diseases, which has attracted much attention in recent years. In this review, we summarize the recent findings regarding the effects of zinc on cardiovascular function. We also discuss the importance of maintaining zinc homeostasis in the cardiovascular system and its therapeutic potential as a novel drug target.

## 1. Introduction

**Figure 1:**
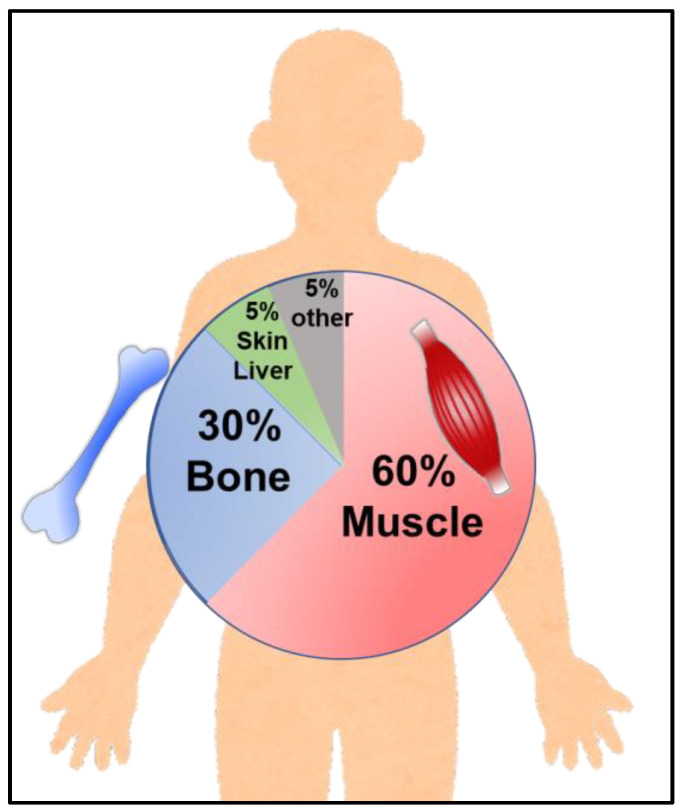
Distribution of zinc in the body. Schematic diagram of zinc distribution in vivo.

Zinc was first reported as an essential trace element important for maintaining health, based on the discovery of “zinc deficiency” more than half a century ago [1]. Since then, various studies have been conducted to reveal zinc-related physiological responses, from both basic principles and clinical points of view. In the last decade, intensive research using genetically modified mice and human genetic analysis have led to outstanding progress in understanding the importance of zinc in terms of in vivo functions and its relationship with human diseases, attracting renewed attention. Zinc regulates various physiological responses, including growth, differentiation, maturation, and immune responses. Approximately 60% of the zinc in the body is stored in skeletal muscles, 30% in bones, and 5% in the skin and liver (Figure 1). Approximately 10% of the proteins in the human body exert their functions by binding with zinc and controlling various physiological responses at the molecular level [2,3,4,5]. Therefore, proper regulation of zinc levels in vivo is vital for maintaining physiological functions and health. Prolonged zinc deficiency and low zinc status caused by inadequate dietary zinc intake can give rise to a plethora of detrimental health consequences. Low zinc status can lead to various symptoms such as parageusia, skin disorder, and growth retardation, highlighting the importance of zinc homeostasis in maintaining vital activities [6]. Hence, ensuring adequate zinc intake through diet or supplementation is critical for averting deleterious health consequences associated with low zinc status.

**Figure 2:**
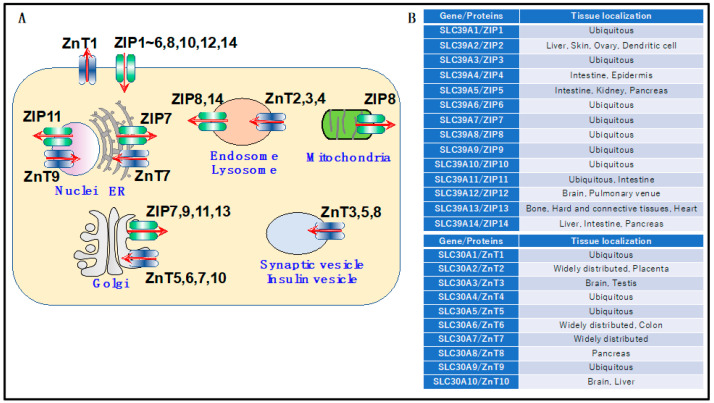
Schematic diagram of zinc transporter distribution in the cell (**A**) and in the tissues (**B**). Zinc transporters are expressed in organelles and cell membranes of various tissues. To maintain zinc levels in the cells, ZIP family members increase intracytoplasmic zinc concentration, and ZnT family members decrease intracytoplasmic zinc concentration.

Intracellularly, zinc binds to metallothionein (MT), a zinc-binding protein distributed in the cytosol and organelles. MT is involved in regulating oxidative stress, such as reactive oxygen species (ROS), and inflammatory responses and thus plays a part in the mechanism of zinc homeostasis in vivo. In addition to MT, one of the main components of the molecular mechanisms regulating zinc homeostasis is the zinc transporter family. This family includes Solute Carrier Family 39A (SLC39A)/Zrt- and Irt-like proteins (ZIPs) and Solute Carrier Family 30A (SLC30A)/Zinc transporters (ZnTs). There are 14 types of ZIPs and 10 types of ZnTs, which are known to be localized on the membranes of intracellular organelles in the various tissues (Figure 2A,B). Both SLC39A/ZIPs and SLC30A/ZnTs are capable of transporting zinc. However, ZIPs augment cytosolic zinc concentration, whereas ZnTs decrease this. This is thought to preserve zinc homeostasis within an organism. In previous studies on zinc transporters, analyses utilizing genetically modified mice have been conducted, and the patterns of expression and physiological functions in tissues have been examined. These studies have revealed that zinc transported by the zinc transporter regulates intracellular signal transduction as a “zinc signal” and that abnormalities in zinc signaling are closely linked to the onset and mechanisms of disease. Zinc signaling has received considerable attention as a potential target for drug discovery. Furthermore, in addition to zinc transporters, recent reports have identified other cell membrane proteins that contribute to the regulation of zinc homeostasis. These molecules are expected to be valuable from the perspective of prevention and treatment, as they are attractive molecules involved in zinc regulation.

Cardiovascular diseases (CVDs) are the leading cause of death worldwide and includes a wide range of conditions, such as coronary artery disease, heart failure (HF), stroke, and hypertension, all of which are major causes of disability and death [7]. Therefore, the treatment of CVDs is of critical importance. The onset of CVDs is typically induced by lifestyle factors such as a Western diet, lower physical activity, and smoking habits, as well as some genetic disorders and conditions resulting from high blood pressure and hyperlipidemia. As described in the section below, epidemiological studies have suggested a potential link between low serum zinc levels and increased CVD risk. The precise mechanisms underlying zinc-related cardiovascular function have not been well investigated. However, recent studies have reported the molecular mechanisms underlying the relationship between zinc and CVDs.

This review summarizes epidemiological studies on the relationship between serum/plasma zinc levels or supplementation and CVDs. It also introduces the intracellular molecular mechanisms regulated by zinc ions related to cardiovascular functions. As potential therapeutic targets, we describe the involvement of SLC39A and SLC30A zinc transporter families and other membrane proteins regulating zinc homeostasis, along with recent reports, including our research, in terms of their physiological and pathophysiological roles in the cardiovascular system.

## 2. Epidemiological Studies of Zinc Level and CVDs

Numerous analyses of the relationship between zinc and type2 diabetes and CVDs in humans have been reported (Table 1 and Table 2). The results may not necessarily indicate a consistent trend and may be influenced by factors such as the number and demographics of the study population, sex, and conditions used to categorize the study groups. There is much information about the beneficial effects of zinc supplementation on health, not only in the nutritional sciences but also in the clinical situation. However, the effects of zinc supplementation or zinc intake on the risk of cardiovascular diseases is debatable in epidemiological studies, although it has been suggested that zinc intake may contribute to a decreased risk of cardiovascular diseases resulting from type 2 diabetes due to its association with a decreased risk of type 2 diabetes (Table 1). Nevertheless, the meta-analysis of the studies of the effect of zinc intervention on risk for CVDs reported that the dose and duration of zinc supplementation contributed to the difference in benefits regarding risk factors for CVDs. Long-duration and low-dose supplementation was found to be more beneficial than short-duration (less than 12 weeks) and high-dose (more than 25 mg/day) zinc supplementation [8]. Another meta-analysis of randomized controlled trials of the effect of zinc supplementation on serum zinc concentration in prepubertal children showed that zinc supplementation had a positive effect on the increase of serum zinc concentration [9]. In a randomized, double-blind, placebo-controlled trial of the effect of zinc supplementation on serum zinc concentration in 53 elderly individuals with low serum zinc (<70 μg/dL), the difference in the mean change in serum zinc between the 30 mg/day zinc supplementation group and the placebo group was found to be significant [10]. Interestingly, the serum zinc levels of the participants with low serum zinc (<60 μg/dL) were not increased to more than 70 μg/dL. Additionally, many studies have demonstrated an association between serum zinc levels and CVD risk, with particularly low serum zinc levels being associated with an increased risk of CVD onset (Table 2). Some reports have also suggested that maintaining high serum zinc levels may be associated with a reduced risk of CVD. As a result, serum zinc levels are considered valuable biomarkers. However, there are several limitations that need to be taken into account when using serum zinc levels as a biomarker, including diurnal variation and discrepant values, depending on the timing of blood collection for testing [11,12]. Although zinc supplementation is commonly used to treat zinc deficiency, it is not always effective in fully restoring serum zinc levels in some patients. Accordingly, the use of serum zinc levels as a biomarker in cardiovascular disease is deemed valuable; however, this suggests that the relationship between zinc supplementation and serum zinc levels is complex and not necessarily straightforward, and highlights the need for further research to better understand the underlying mechanisms regulating zinc homeostasis, including dynamics of zinc distribution intracellular and extracellular regions, and the role of zinc transporters and other molecules, as described in later sections (Section 3, Section 4 and Section 5).

## 3. Zinc-Mediated Intracellular Processes Related on Cardiovascular Functions

### 3.1. Oxidative Stress

**Figure 3:**
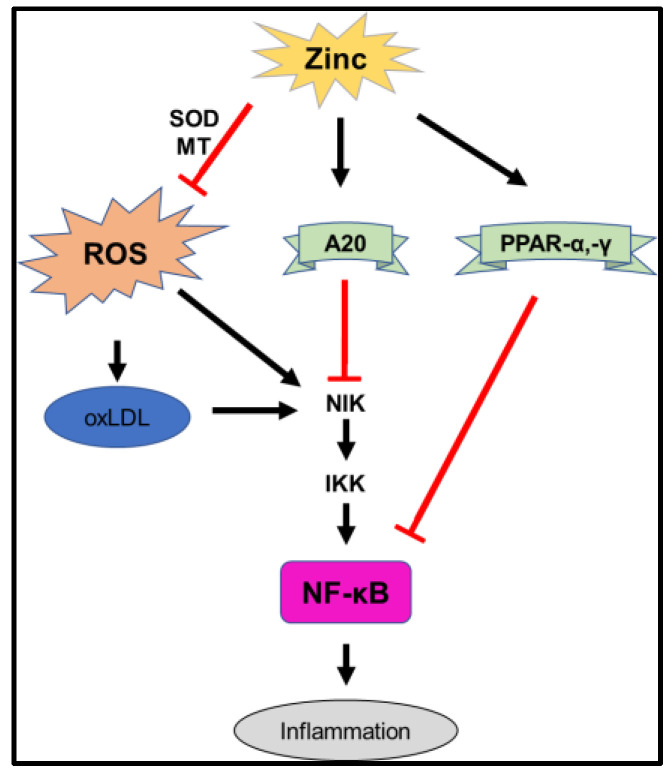
Mechanism of inflammation inhibited by zinc. Schematic diagram of zinc-mediated suppression of inflammatory pathways. Arrows indicate activation or signaling, and red lines indicate inhibition. MTs, whose expression is upregulated by zinc, suppress ROS production. Since ROS and NIK both activate NF-κB, zinc suppresses inflammation by inhibiting NF-κB-mediated pathways and plays a role in suppressing inflammation by inhibiting the NF-κB-mediated pathway. NIK: NF-κB-inducing kinase. IKK: Inhibitor of κB kinase. PPAR: Peroxisome Proliferator-Activated Receptor.

Multiple reports have highlighted the involvement of zinc in CVD, with one of the molecular mechanisms underlying CVD being related to inflammation via oxidative stress with ROS. Activation of the NF-κB signaling pathway by ROS leads to the induction of inflammatory cytokines (IL-1β, IL-6, IL-8, TNF-α, and MCP-1) and inflammation-related enzymes (iNOS and COX2). Zinc acts as a cofactor for superoxide dismutase (SOD) through the production of MT, which inhibits ROS production. Additionally, zinc inhibits the activation of the NF-κB pathway through the activation of PPAR-α, PPAR-γ, and A20. Zinc also inhibits inflammation (Figure 3).

### 3.2. Metallothionein (MT)

MT is a cysteine-rich, low-molecular-weight protein found in all the cells of various animal and plant tissues. It plays a crucial role in regulating the homeostasis of both essential and toxic metals such as zinc, copper and cadmium. Depending on the bound metal ions, MTs exhibit different stoichiometries, showing the two domains in a simplified geometry. Four isoforms of MT are reported, of which MT-1 and MT-2, that can bind up to seven zinc ions in a MT molecule, are mainly expressed in the liver and kidney. MT generally has antioxidant, detoxification, and cyto-protection properties in various stress responses [37]. MT also affects cellular processes such as gene expression, differentiation, apoptosis and proliferation. MT expression is induced and enhanced in various tissues by physiological mediators and various stresses, including inflammatory stimuli. It is considered a biomarker for metal exposure and is involved in the pathogenesis of various diseases, including liver cirrhosis, cancer, neurodegenerative disorders, and CVDs. Ceylan-Isik et al. demonstrated that the impairment of cardiac function observed in an LPS-induced mouse sepsis model was restored in a sepsis model using MT-overexpressing mice, which showed lower ROS production [38]. Hu et al. similarly found that myocardial contractility was reduced in mice exposed to secondhand cigarette smoke due to oxidative stress and mitochondrial damage. However, this reduction was not observed in MT-overexpressing mice owing to the inhibitory effect of MT on ROS production and mitochondrial damage [39]. Additionally, Cai et al. observed that, in STZ-treated diabetic model mice, cardiomyocyte apoptosis was induced by oxidative stress-mediated caspase activation via mitochondrial cytochrome c release, but this phenomenon was not observed in mice with myocardial-specific MT overexpression [40]. Furthermore, Huang et al. reported that MT overexpression in cardiomyocytes had a protective effect on diabetic cardiomyopathy in the serine/threonine-protein kinases’ Akt2 gene (*Akt2*)-KO mice, which was attenuated by ERK1/2 phosphorylation-regulated glucose transport and metabolism [41]. These reports indicate that MT may play a crucial role in regulating cardiac function by mitigating cardiac stress caused by oxidative stress and inflammatory processes. Further research is required to elucidate how the aberrant intracellular zinc level caused by the expression changes of zinc transporters might affect the binding of zinc to MT. There may be a relationship between specific zinc transporters and MTs that regulate cardiovascular functions.

In the next section, we will introduce the specific zinc transporter functions that regulate cardiovascular functions, together with our recent research.

## 4. Zinc Transporters Regulating Cardiovascular Functions

There are some reports regarding the slc30a and slc39a zinc transporter families that regulate cardiovascular function. Four zinc transporters, reported as key molecules that can modulate cardiovascular function, are introduced in this section.

### 4.1. ZnT1

ZnT1 is ubiquitously expressed on plasma membranes and strictly regulates intracellular zinc levels by exporting zinc from the cytosol [42]. *ZnT1*-KO mice show embryonic lethality, and ZnT1 modulated intracellular zinc has been reported to be involved in cancer progression [43]. In terms of the relationship with cardiovascular function, Beharier et al. reported the relationship between cardiac L-type calcium channels (LTCC) and ZnT1 function. The inhibitory effect of ZnT1 on steady-state current-voltage (I-V) was observed in Xenopus oocytes expressing both ZnT1 and LTCC. ZnT1 expression increased in the atria of rats with rapid-pacing treatment and in patients with atrial fibrillation [44]. Considered together, these results indicate that the ZnT1 function is related to the modulation of LTCC function; however, the precise molecular mechanisms by which specific target molecules of ZnT1 or ZnT1-mediated zinc function need to be investigated.

### 4.2. ZnT-5

ZnT5 is mainly expressed in the Golgi apparatus. Inoue et al. reported that *ZnT5*-KO mice showed reduced bone density owing to osteoblast immaturity and growth retardation. In particular, male *ZnT5*-KO mice showed sudden death due to brady-arrhythmias, which may be caused by the downregulation of genes related to immediate-early response factors and heat shock proteins [45]. Since ZIP13 expressed in the Golgi apparatus of the cardiomyocytes is involved in the cardiac functions (see the Section 4.5 in detail), lack of ZnT5 expression might induce the impairment of zinc level in the Golgi apparatus leading to cardiac dysfunctions.

### 4.3. ZnT7 and ZIP7

ZnT7 and ZIP7 are expressed in the endoplasmic reticulum and are involved in the regulation of ER zinc levels during ER stresses [46,47]. Tuncay et al. reported that ZnT7 and ZIP7 were expressed in the mitochondria, and their expression was decreased in cardiomyocytes treated with hyperglycemia and doxorubicin. Hyperglycemic stimulation of cardiomyocytes increased intra-mitochondrial zinc and ROS levels, which would induce mitochondrial dysfunctions [48]. Nolin et al. reported a ZIP7 inhibitor (NVS-ZP7-4) that modulates ER zinc levels and Notch signaling [49]. Therefore, regulating ZIP7 function in cardiomyocytes may be a potential therapeutic strategy for treating CVDs.

### 4.4. ZIP2

ZIP2 expression in the heart is quite low under normal conditions; however, it is upregulated in response to ischemia/reperfusion injury [50]. *Zip2*-KO mice show abnormal embryonic development. ZIP2-modulated intracellular zinc is involved in keratinocyte differentiation [51,52]. Regarding its relation with cardiovascular functions, Du et al. reported that ZIP2, a known zinc importer, has a cardioprotective effect on myocardial ischemia/reperfusion (I/R) injury [50]. ZIP2 is upregulated during reperfusion in the mouse heart and is induced by STAT3 phosphorylation in response to I/R injury. The STAT3-phosphorylation-induced protective effect on I/R injury was reduced in *Zip2*-KO mice. In addition, I/R injury-induced STAT3 phosphorylation is inhibited by zinc chloride. Zhao et al. also reported that, as the expression regulation of ZIP2 in I/R injury, the NADPH oxidase isoform (NOX2) cytosolic factor p67^phox^ and p67^phox^-derived ROS production was upregulated in I/R injury and operated ZIP2 expression via STAT3 phosphorylation. In contrast, the I/R-induced loss of zinc content in the heart upregulated p67^phox^ expression [53].

These findings indicate that ZIP2-mediated zinc homeostasis and regulation of STAT3 expression are critical protective mechanisms against I/R injury.

### 4.5. ZIP8

ZIP8 is widely expressed in the body on plasma membranes, and transports zinc and manganese into the cytosol. In humans, autosomal-recessive intellectual disability with cerebellar atrophy syndrome is reported to be partly induced by a mutation in the ZIP8 gene [54]. In addition, Crohn’s disease and gut microbiome composition were affected by the ZIP8 SNP. In terms of the relationship with cardiovascular functions, Lin et al. reported a relationship between ZIP8 and cardiac ventricular compaction. ZIP8, expressed in endothelial cells in the developing mouse heart, plays a vital role in maintaining cellular zinc levels. The accumulation of extracellular matrix (ECM) and the reduction of several disintegrin and metalloproteinase with thrombospondin motifs (ADAMTS) that can degrade ECM were observed in heart tissue derived from *Zip8*-KO mice [55].

### 4.6. ZIP13

**Figure 4:**
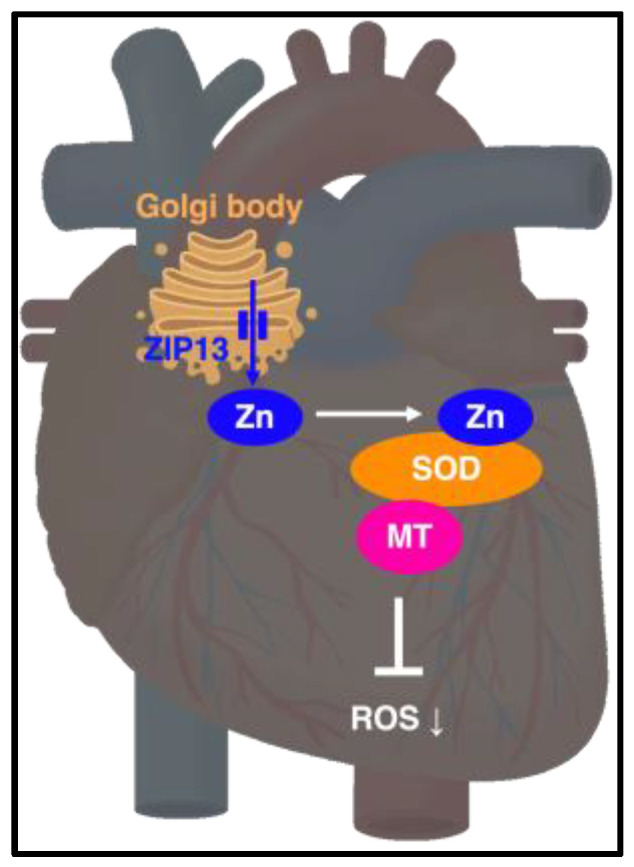
ZIP13 indicates the maintenance of cardiovascular homeostasis by resolving inflammation and stress response. ZIP13 plays a role in cardiovascular homeostasis by resolving inflammatory and stress responses. ZIP13 significantly affects cardiac physiology; therefore, it is a potential therapeutic target for CVD.

ZIP13 is expressed in the Golgi apparatus membranes of the cells derived from mesenchymal stem cells. A genetic mutation in the ZIP13 gene caused Spondylocheiro-dysplastic Ehlers–Danlos syndrome (EDS-SPD3), which showed growth retardation, thin, hyper-elastic skin, hypermobile small joints, and muscle atrophy [56,57,58]. Several studies have reported the role of ZIP13 in cardiovascular function. Wang et al. reported that ischemia/reperfusion (I/R) injury with reperfusion and reoxygenation induced the downregulation of ZIP13 expression in the heart [59]. *Zip13*-KO mice exhibit increased phosphorylation of Ca^2+^-calmodulin-dependent protein kinase (CaMKII), leading to cardiac remodeling and arrhythmia. Furthermore, the hearts of *Zip13*-KO mice displayed increased levels of ROS, mitochondrial calcium, and morphological abnormalities. Hara et al. reported that doxorubicin-induced cardiotoxicity results in the upregulation of ZIP13 expression in the heart. *Zip13*-KO neonate-derived primary cardiomyocytes and *Zip13*-KO mice displayed aberrant heartbeats, similar to arrhythmia. Additionally, the hearts of *Zip13*-KO mice exhibited upregulation of inflammatory genes and Golgi stress-induced genes compared to wild-type mice (Figure 4). Hirose et al. reported that aortic fragility is characterized by abnormalities in cellular components and the morphology of elastic components was observed in the thoracic aorta of *Zip13*-KO mice [60,61]. Traylor et al. conducted a genome-wide association analysis of patients diagnosed with lacunar stroke. GWAS data analysis predicted the possibility of lacunar stroke risk and gene pathways, including ZIP13 (SPI1-ZIP13-PSMC3-RAPSN) [62].

These reports of cardiovascular functions in the slc30a and slc39a families suggest that the regulation of zinc homeostasis in the heart tissues by zinc transporters is crucial for maintaining cardiac function, and the modulation of zinc transporter expression and function may serve as a beneficial therapeutic target for CVDs.

## 5. Membrane Proteins Regulating Zinc Homeostasis in Calcium Homeostasis (Other Membrane Proteins Involved in the Regulation of Zinc Homeostasis)

### 5.1. G-Protein Coupled Receptor 39 (GPR39)

**Figure 5:**
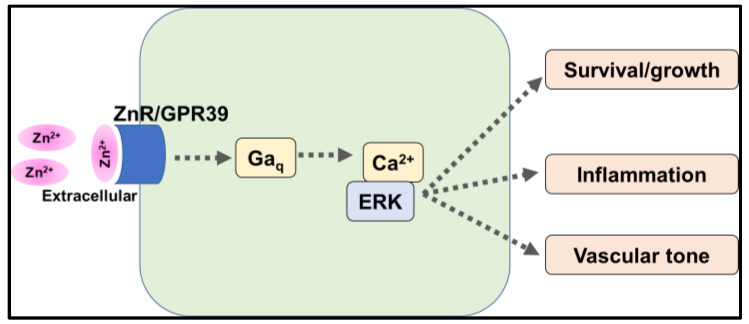
Pathway of intracellular activity by zinc signal via ZnR/GPR39. Zinc signaling via ZnR/GPR39 activates MAPK/ERK via Gαq, which is involved in cell survival and proliferation, angiogenesis, inflammatory responses, and vascular tone.

Zhu et al. demonstrated that intracellular signaling via GPR39, stimulated by the extracellular physiological level of zinc, was involved in vascular endothelial cell proliferation, survival, angiogenesis, and inflammatory responses via activation of Gaq-PLC and PI3-ERK (Figure 5**)**. These effects were not observed in *Gpr39*-KO mice-derived endothelial cells [63]. Meda et al. reported that GPR39 expression was increased in the endothelial cells of patients with type 2 diabetes. Both GPR39-overexpression and agonist stimulation impaired endothelial function through the activation of SHH pathways. *Gpr39*-KO mice showed a protective phenotype in which tissue necrosis and mal-vascularization were not observed in diabetic models [64]. Liao et al. demonstrated that GPR39 inhibited AMPK signaling with mTOR and S6k1 activation, which induced the activation of de novo protein synthesis, leading to cardiac hypertrophy in mice. GPR39 overexpression in neonatal cardiomyocytes exacerbates angiotensin II-induced cardiac hypertrophy [65].

Although the agonistic effect of zinc on GPR39 activation under physiological conditions is still debatable, because GPR39 would show constitutive activity even without zinc stimulation, these findings indicate that the activation of GPR39 is involved in the pathophysiological conditions both in cardiomyocytes and endothelial cells, suggesting that the regulation of GPR39 expression and function, especially the agent with an inhibitory effect on GPR39, might have beneficial effects on CVDs.

### 5.2. Transient Receptor Potential Ankyrin 1 (TRPA1)

**Figure 6:**
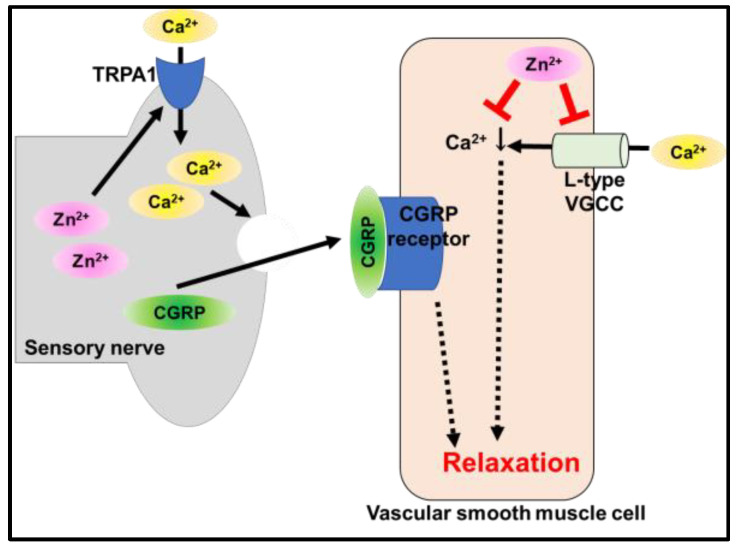
Mechanism of zinc-mediated vasorelaxation. Sensory nerve zinc promotes Ca^2+^ influx in transient receptor potential ankyrin 1 (TRPA1), and the influx of Ca^2+^ causes calcitonin gene-related peptide (CGRP) release. The released CGRP binds to the CGRP receptors in smooth muscles, causing smooth muscle relaxation. On the other hand, smooth-muscle zinc causes smooth muscle relaxation by inhibiting Ca^2+^ influx through L-type voltage-gated calcium channels (VGCC).

Betrie et al. demonstrated the effect of changing cytoplasmic zinc levels in blood vessels and smooth muscles. An increase in cytoplasmic zinc using a zinc ionophore showed a vasorelaxation effect; however, the chelation of cytoplasmic zinc increased the contraction of blood vessels and smooth muscles (Figure 6). They suggested that the effect of elevated cytoplasmic zinc levels stimulated TRPA1 activation, followed by the modulation of calcitonin gene-related peptide (CGRP) signaling in the sensory nerves, inhibiting voltage-gated calcium channels in vascular smooth muscle cells [66].

These findings provide clear evidence that an increase in cytoplasmic zinc levels could be a potential therapeutic strategy for CVDs, although it is vital to understand the systemic molecular mechanisms of zinc homeostasis.

### 5.3. Transient Receptor Potential Cation Channel 6 (TRPC6)

Gibon et al. reported that TRPC6 channels are involved in the regulation of intracellular zinc levels. HEK293 cells expressing TRPC6 and E13 cortical neurons showed increased intracellular zinc levels when treated with the TRPC6 channel activator SAG [67]. These findings indicate that, not only the slc30a and slc39a families, but also other membrane channels act as transporters of zinc ions, and their contribution is definitely important to regulate zinc homeostasis during physiological processes.

**Figure 7:**
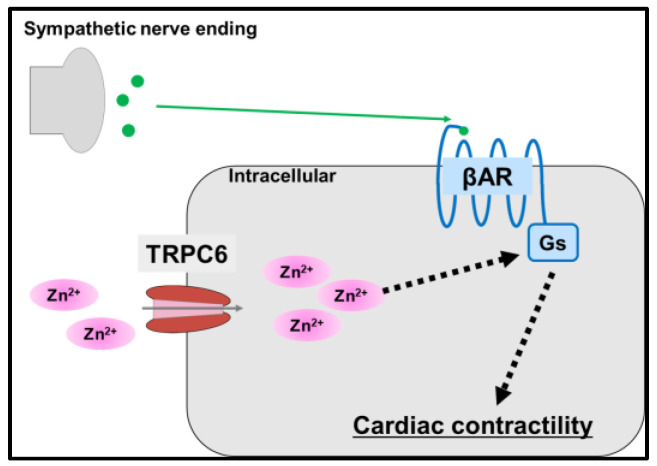
Zinc is involved in cardiac contractility. Schematic of the signaling pathway via TRPC6. Solid arrows represent mass transfer, dashed arrows represent signal transduction, and intracellular zinc influx via TRPC6 enhances β-adrenergic receptor (AR)-mediated Gs signaling, resulting in enhanced myocardial contractility.

Oda et al. also demonstrated that activation of TRPC6 channels modulates zinc levels in cardiomyocytes [68]. Isoproterenol-induced cAMP production was enhanced by the stimulation of norepinephrine, which was reduced in *Trpc6* knockdown cells, and the cells derived from the mice with the TRPC6 mutant gene in which TRPC6 mediated zinc influx induced by norepinephrine stimulation was abrogated. Furthermore, treatment with the TRPC6 activator PPZ2 in HF model mice with transverse aortic constriction operation increased zinc concentration, which was decreased in *Trpc6* KO mice. In addition, PPZ2 treatment ameliorated the reduced ejection fraction observed in myocardial infarction. As TRPC6 channel function in cardiomyocytes remains unclear, these findings suggest the possibility of developing novel therapeutic strategies targeting TRPC6 channel modulation for CVDs (Figure 7).

### 5.4. Ryanodine Receptor 2 (RyR2) and Mitsugumin 23 (MG23)

**Figure 8:**
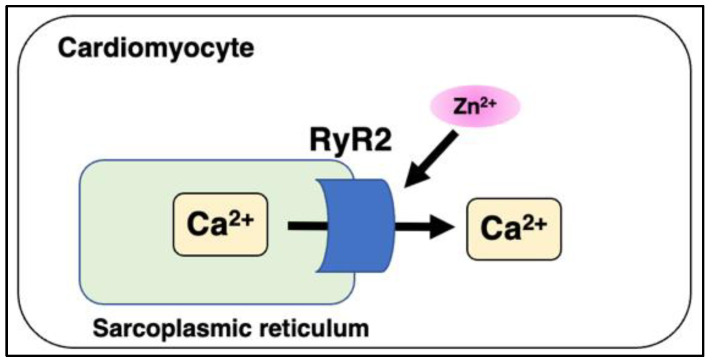
Zinc is involved in ryanodine receptor functions. Schematic of the zinc-mediated function of RyR2 in the SR. Aberrant intracellular zinc levels regulate calcium release via RyR2 and mitsugumin 23 from the SR.

Reilly-O’Donnell reported that aberrant zinc homeostasis impairs cardiac RyR2 and MG23 function in cardiac muscle [69]. In chronic HF, Ca^2+^ leakage from the SR impairs cardiac function; however, the precise molecular mechanisms have not been fully investigated. RyR2 channels located on the sarcoplasmic reticulum (SR) are involved in the cytosolic Ca^2+^ transient. Using the single channel recordings in a voltage-clamp experiment, increased cytosolic zinc levels promoted the activity of RyR2 in the presence of Mg^2+^ (Figure 8). Intracellular zinc level was increased in response to ischemic condition in H9C2 cells, which is linked with the CVDs showing the zinc dys-homeostasis. MG23, expressed on ER and SR, is a voltage-dependent cation channel that showed the increase of its expression under the ischemic conditions in H9C2 cells. An increased zinc level in cytosol also promoted the activity of MG23. These findings indicate that dysregulation of the zinc levels in the cardiac muscle induced by ischemic conditions would affect the Ca^2+^ homeostasis operated by SR [70].

## 6. Therapeutic Perspectives

The zinc transporter family tightly controls the regulation of intracellular zinc homeostasis, and zinc levels are closely monitored in various intracellular compartments, ranging from the cytoplasm to organelles, such as the ER and mitochondria. Our recent study revealed that stress load decreases the expression of ZIP13 in the heart and that ZIP13 dysfunction is associated with cardiac dysfunction, indicating that zinc homeostasis in the heart is crucial for cardiac function, and that compounds that regulate ZIP13 function may serve as novel therapeutic interventions for heart disease. To date, there are no reports about the compounds that regulate ZIP13 function; however, given the association of ZIP13 with cardiovascular regulation, specific compounds targeting ZIP13 are thought to have potential as drug candidates for cardiovascular diseases. In particular, compounds that activate ZIP13 function may be beneficial, as impaired ZIP13 function has been linked to cardiac dysfunction. Moreover, since ZIP13 is expressed in the Golgi apparatus, compounds that regulate Golgi function may have the potential to control ZIP13 expression. We reported elevated expression of Golgi stress markers in the hearts of *Zip13*-KO mice, suggesting a potential linkage between ZIP13 expression regulation and compounds or molecules involved in Golgi function control. These compounds are thought to be useful in the treatment of cardiovascular diseases, and further drug discovery research is anticipated.

**Figure 9:**
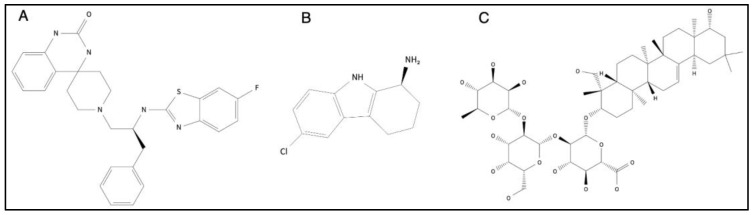
The structure of compounds regulating zinc transporters. (**A**) A ZIP7 inhibitor (NVS-ZP7-4) regulates ER zinc levels and Notch signaling (IC_50_ = 0.13 μM). (**B**) A ZIP8 inhibitor regulates intracellular zinc level (IC_50_ = 17.2 μM). (**C**) Soyasaponin Bb, extracted from soybeans, increased ZIP4 expression.

Previous studies on zinc transporters have demonstrated that each transporter plays a distinct role in physiological responses and disease mechanisms by regulating specific zinc signals. However, challenges and questions remain to be addressed in advancing this research, such as identifying low-molecular-weight compounds that regulate the function of zinc transporters and the mechanisms by which these transporters transport zinc. To date, a few compounds that modulate zinc levels and the expression of zinc transporters have been reported (Figure 9). A ZIP7 inhibitor (NVS-ZP7-4), (S)-1-(2-((6-fluorobenzo[d]thiazol-2-yl)amino)-3-phenylpropyl)-1′H-spiro[piperidine-4,4′-quinazolin]-2′(3′H)-one, 1-[(2S)-2-[(6-fluoro-2-benzothiazolyl)amino]-3-phenylpropyl]-spiro[piperidine-4,4′(1′H)-quinazolin]-2′(3′H)-one, was reported to modulate ER zinc levels and Notch signaling, leading to apoptotic cell death (IC_50_ = 0.13 μM) [49]. A ZIP8 inhibitor, (S)-6-chloro-2,3,4,9-tetrahydro-1H-carbazol-1-amine was identified by Cd^2+^ -uptake assay in the cells transiently expressing ZIP8 (IC_50_ = 17.2 μM). This compound showed cross reactivity to two zinc transporters ZIP2 and ZIP14, and to four membrane proteins, serotonin receptor (5-HT2A), dopamine transporter, serotonin transporter and ERG potassium channel [71]. As food ingredients that can affect the expression of zinc transporter, soyasaponin Bb which is extracted from soybeans decreased zinc-induced mouse ZIP4 (mZIP4) endocytosis and increased the protein expression of both mouse and human ZIP4 and the intracellular zinc levels [72]. These compounds were useful for the potential therapeutic candidates that can either directly or indirectly modulate the functions of zinc transporter. In addition, both the agonists and antagonists of TRPC6 have already been reported, and these compounds can modulate intracellular zinc levels based on the findings described in Section 5.3. These compounds have a significant potential to be candidates for developing therapeutic agents.

In the future, it is imperative to actively investigate the identification of interacting molecular groups. The diagnosis of zinc deficiency and disease prevention based on in vivo zinc levels, particularly serum zinc levels, is logical. However, further understanding is required to extrapolate the serum zinc levels and in vivo zinc status to cellular responses. To that end, in addition to MT, ZnT, and ZIP, a more thorough understanding of the roles of the new molecules involved in zinc homeostasis introduced in this paper is necessary. Further studies are needed to analyze the effects of global genetic variation on these molecules and the relationship between the zinc status in vivo. The effects of drugs on serum zinc levels and the expression of molecules that control zinc kinetics have yet to be fully evaluated. For compounds that can potentially affect serum zinc levels, such as those that form chelates with zinc, their effect can be inferred to some extent from evaluations of clinical information and chemical properties of the compounds. However, there may be drugs that have yet to be identified that affect local zinc kinetics in tissues or specific cells without significantly impacting serum zinc levels. Comprehensive genetic analysis and further research on probes and detection methods for detecting minute changes in local zinc levels will lead to the development of drugs that can help control zinc kinetics.

Controlling serum zinc levels through zinc supplementation or administering zinc preparations as pharmaceuticals is highly beneficial. Furthermore, research on specific compounds and food ingredients that affect molecules involved in zinc homeostasis may serve as promising starting points for drug discovery.

## 7. Conclusions

This review summarizes recent findings on the importance of zinc in epidemiological and molecular biology studies. A growing body of evidence indicates the significance of serum zinc levels and their homeostatic mechanisms in relation to cardiovascular function, both in physiological and pathophysiological conditions. Epidemiological studies suggested that low plasma(serum) zinc levels increase the risk of developing cardiovascular diseases, while supplementation of zinc may decrease this risk, which indicate the importance of appropriate zinc management in disease prevention. Additionally, the molecular mechanisms of zinc-mediated regulation of cardiac function, including zinc transporters and calcium signaling, have been reported. Hence, it is crucial to understand how these molecular mechanisms affect serum zinc levels. Further, the specific inhibitors of zinc transporters are useful as experimental tools for understanding the pathophysiological molecular mechanisms of CVDs, as well as the potential drug candidates.

Taken together, although further intensive research is expected, zinc homeostasis is a promising therapeutic target for the prevention and treatment of cardiovascular diseases.

## Figures and Tables

**Table 1:** Epidemiological studies of the effect of zinc intake on the risk of type2 diabetes and CVDs.

Zinc Status	Population	Baseline Age (Year)	Sex (%)	Follow-Up (Year)	No. of Cases	Outcome	References	Prospective Risk of CVD or T2D
Zinc supplementation	Health professionals, excluded participants with cancer, MI and CVD	40–75	Male	12	39,633	Risk of CVD	Al-Delaimy et al., 2004 [13]	No association
Zinc intake	Postmenopausal women without having angina, heart disease or heart attack	55–69	Female	18	34,492	CVD mortality	Lee, D.H., et al., 2005 [14]	↓ risk
Zinc intake	Nurses free of diabetes, cancer or CVD at baseline	33–60	Female	24	82,297	Risk of T2D	Q. Sun et al., 2009 [15]	↓ risk
Zinc supplementation	Mostly postmenopausal women	55–69	Female	10	38,772	CVD mortality	J. Mursu et al., 2011 [16]	No association
Zinc supplementation	NIH-AARP cohort (Frequent use of any multivitamins including zinc)	50–71	Female (64.5%)	10	232,007	Risk of T2D	Y. Song et al., 2011 [17]	No association
Zinc intake	Population-based sample, free of clinical CVD	61.8 ± 10.3	-	10	5285	Risk of CVD	M.C. Otto et al., 2012 [18]	↓ risk
Zinc intake	Women	45–50	Female	6	8921	Risk of T2D	K.P. Vashum et al., 2013 [19]	No association
Zinc intake	African American and Caucasian men and women	27.03 ± 3.61	Female (52.5%)	23	3960	Risk of T2D	J.S. Park et al., 2016 [20]	↓ risk

**Table 2:** Epidemiological studies of the relationship between zinc level and the risk of CVDs.

Zinc Status	Population	Baseline Age (Year)	Sex (%)	Follow-Up (Year)	No. of Cases	Outcome	References	Prospective Risk of CVD
Serum zinc level	A Dutch prospective follow-up study	N/A	N/A	6–9	10,658	Risk of death from CVD	F.J. Kok et al., 1988 [21]	No association
Serum zinc level	Rural and urban Indian populations	26–65	Female (53%)	-	314	Coronary artery disease (CAD)	R.B. Singh et al., 1997 [22]	↓ risk
Plasma/serum zinc level	Community-living age ≥ 65 years	≥65	Female (47%)	13	344	serum zinc level and vascular mortality	J. Marniemi et al. 1998 [23]	No association
Plasma/serum zinc level	Men aged ≥ 30 year	30–60	Male	20	4035	diabetes, and CVD history	N. Leone et al., 2006 [24]	No association
Serum zinc/urine zinc	Saudi male subjects	N/A	Male	-	260	CVD and serum zinc	E.M. Alissa et al., 2006 [25]	Association
Serum zinc	Iranian subjects undergoing coronary angiography	N/A	Female (41%)	-	114	CAD: lower serum zinc	S.M. Kazemi-Bajestani et al., 2007 [26]	Association
Plasma/serum zinc level	Patients with Type 2 DM	45–64	Female (45%)	7	1050	Higher baseline serum zinc level was associated with reduction in risk of CHD death.	M. Soinio et al., 2007 [27]	↓ risk
Plasma/serum zinc level	German ancestry who was referred to coronary angiography	>60 (median)	Female (30%)	7.75	3274	Increased CVD mortality and serum zinc.	S. Pilz et al., 2009 [28]	Association
Plasma/serum zinc level	Community-living participants Age ≥ 65 years	65–99	Female (49%)	14	741	Risk of vascular disease mortality	C.J. Bates et al., 2011 [29]	Association
Plasma/serum zinc level	A group of 21 subjects free of symptoms, signs and objective evidence of HF	69 ± 11	Male (71%)	6	125	HF and patients with left ventricular diastolic function	I. Alexanian et al., 2014 [30]	No association
Plasm zinc	Subjects with uneventful cardiovascular history and without anti-inflammatory treatments	80–102	-	-	201	coronary calcium score,	R.C. De Paula et al., 2014 [31]	No association
Serum zinc level	2886 subjects from 41 case-control studies (Meta-analysis of 13 articles)	-	-	-	2886	Myocardial infarction and serum zinc	B. Liu et al., 2015 [32]	Association
Plasma/ Serum zinc	Summarize prospective cohort studies from 14 articles	-	-	12.4	91,708	CVD and zinc status	A. Chu et al., 2016 [33]	Association
Plasma/serum zinc level	Finnish men	42–60	Male	20	2220	Incidence of T2D and serum zinc and	T. Yary et al., 2016 [34]	Association
Plasma/serum zinc level	Left ventricular hypertrophy (LVH) patients, especially in the eccentric LVH and concentric LVH patients	61.8 ± 13.2	Female (44%)	-	519	left ventricular hypertrophy (LVH)	L. Huang et al., 2017 [35]	↓ risk
Serum zinc level	1453 subjects from 27 case-control studies (Meta-analysis of 12 articles)	-	-	-	1453	HF and low serum zinc	X. Yu et al., 2018 [36]	Association
